# Aero-Engine Ablation Defect Detection with Improved CLR-YOLOv11 Algorithm

**DOI:** 10.3390/s25216574

**Published:** 2025-10-25

**Authors:** Yi Liu, Jiatian Liu, Yaxi Xu, Qiang Fu, Jide Qian, Xin Wang

**Affiliations:** 1 Key Laboratory for Civil Aviation Data Governance and Decision Optimization, Civil Aviation Management Institute of China, Beijing 100102, China; liuyi@camic.cn; 2 School of Computer Science, Civil Aviation Flight University of China, Guanghan 618307, China; liujiat@cafuc.edu.cn (J.L.); wangxin@cafuc.edu.cn (X.W.); 3 School of Economics and Management, Civil Aviation Flight University of China, Guanghan 618307, China; 4 Key Laboratory of Flight Techniques and Flight Safety, Civil Aviation Flight University of China, Guanghan 618307, China

**Keywords:** aero-engine, YOLOv11, rotated detection, context-guided mechanism, large-kernel convolutional attention

## Abstract

Aero-engine ablation detection is a critical task in aircraft health management, yet existing rotation-based object detection methods often face challenges of high computational complexity and insufficient local feature extraction. This paper proposes an improved YOLOv11 algorithm incorporating Context-guided Large-kernel attention and Rotated detection head, called CLR-YOLOv11. The model achieves synergistic improvement in both detection efficiency and accuracy through dual structural optimization, with its innovations primarily embodied in the following three tightly coupled strategies: (1) Targeted Data Preprocessing Pipeline Design: To address challenges such as limited sample size, low overall image brightness, and noise interference, we designed an ordered data augmentation and normalization pipeline. This pipeline is not a mere stacking of techniques but strategically enhances sample diversity through geometric transformations (random flipping, rotation), hybrid augmentations (Mixup, Mosaic), and pixel-value transformations (histogram equalization, Gaussian filtering). All processed images subsequently undergo Z-Score normalization. This order-aware pipeline design effectively improves the quality, diversity, and consistency of the input data. (2) Context-Guided Feature Fusion Mechanism: To overcome the limitations of traditional Convolutional Neural Networks in modeling long-range contextual dependencies between ablation areas and surrounding structures, we replaced the original C3k2 layer with the C3K2CG module. This module adaptively fuses local textural details with global semantic information through a context-guided mechanism, enabling the model to more accurately understand the gradual boundaries and spatial context of ablation regions. (3) Efficiency-Oriented Large-Kernel Attention Optimization: To expand the receptive field while strictly controlling the additional computational overhead introduced by rotated detection, we replaced the C2PSA module with the C2PSLA module. By employing large-kernel decomposition and a spatial selective focusing strategy, this module significantly reduces computational load while maintaining multi-scale feature perception capability, ensuring the model meets the demands of high real-time applications. Experiments on a self-built aero-engine ablation dataset demonstrate that the improved model achieves 78.5% mAP@0.5:0.95, representing a 4.2% improvement over the YOLOv11-obb which model without the specialized data augmentation. This study provides an effective solution for high-precision real-time aviation inspection tasks.

## 1. Introduction

Aero-engines, as the core power components of modern aircraft, operate in what can be considered the most extreme industrial environments, making them among the most prone to wear and damage [1]. Subjected to prolonged operation under severe conditions including high temperatures, high pressures, high rotational speeds, and alternating loads [2], critical hot-end components (such as turbine blades and combustion chamber walls) inevitably experience various forms of ablative damage. Undetected or untreated, such damage may lead to gradual engine performance degradation at best, or complete engine failure at worst, posing serious threats to flight safety and potentially causing catastrophic accidents. Consequently, regular inspection and maintenance of aero-engines are of paramount importance. Traditional aero-engine inspection primarily relies on manual examination by trained technicians using probes to assess internal components. However, this approach presents several limitations: First, inspection results are highly dependent on the technician’s expertise, making them susceptible to subjective judgment. Second, the complex internal structure of engines combined with poor lighting conditions and the potential microscopic scale of some ablation damage (like microcracks, early coating failure, and shallow ablation pits) can easily lead to oversight. These undetected micro-damages may progressively expand during subsequent operation, eventually developing into major safety hazards. Third, manual inspection processes are time-consuming and inefficient, struggling to meet modern aviation industry’s demand for high-efficiency maintenance. These limitations have driven the need for more intelligent and efficient detection methods to enhance the reliability and efficiency of aero-engine maintenance.

With the rapid development of computer vision technology, machine learning and deep learning are being increasingly applied to aircraft visual inspection [3]. However, when general object detection frameworks are directly transferred to the specialized scenario of aero-engine ablation detection, they face numerous fundamental challenges. From a visual feature perspective, ablation regions exhibit unique morphological characteristics: the transition zone between heat-affected areas and normal materials forms gradient boundaries, where the edges display fractal features with multi-scale properties while simultaneously showing compositional gradient variations caused by elemental diffusion [4,5,6]. Currently, Chen et al. [7] proposed a hybrid model based on BP neural networks and genetic algorithms that optimizes network connection weights through the BP algorithm while adaptively adjusting model parameters using genetic algorithms, achieving automatic identification of blade tip curl, corrosion, cracks, and tear damage in aero-engine borescope images. Shen et al. [8] employed a fully convolutional network (FCN) to perform semantic segmentation of defects in borescope images, improving damage region localization accuracy through multi-scale feature fusion, ultimately achieving 90% classification accuracy in experiments. The Transformer architecture has also demonstrated notable application potential in aero-engine blade detection tasks. Zhang et al. [9] integrated their proposed Adaptive Context-Aware Weighting (ACAW) module and a Multi-scale Feature Fusion (MFF) module into the Detection Transformer (DETR) framework, achieving an mAP@0.5 of 92.8% in blade defect detection. On the other hand, Mohammadi et al. [10] compared the performance of VGG19 and the Data-efficient Image Transformer (DeiT) in detecting defects in aero-engine components, finding that the Transformer-based approach showcased advantages in both detection accuracy and inference efficiency. These results indicate that Transformer-based architectures hold significant value for further exploration in enhancing aerospace defect detection performance. Existing mainstream detection algorithms generally face a dilemma where some methods achieve high detection accuracy but suffer from insufficient real-time performance due to excessive computational complexity, while other algorithms meet high-speed detection requirements but demonstrate limited accuracy due to constrained feature extraction capabilities.

**Figure 1:**
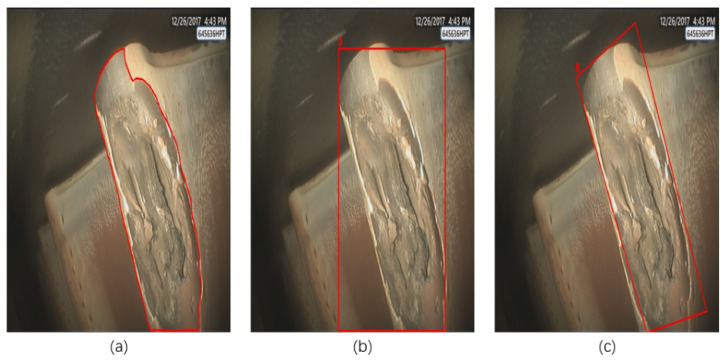
The red box in the figure marks the ablation area. (**a**) Original ablation region image; (**b**) annotation results using conventional horizontal bounding boxes; (**c**) annotation results using rotated bounding boxes.

The introduction of rotation-based object detection frameworks has partially mitigated the issue of localization inaccuracy. As illustrated in Figure 1, we present a comparative analysis of three detection results: (a) the original image of the ablation region, (b) annotation results using conventional horizontal bounding boxes, and (c) annotation results using rotated bounding boxes. The visual comparison clearly demonstrates that rotated bounding boxes can more precisely conform to the actual contours of ablation regions. Experimental results further indicate that rotated bounding boxes reduce approximately 30% of background noise interference compared to traditional horizontal bounding boxes. This improvement enables the model to learn more discriminative feature representations, thereby significantly enhancing detection performance. However, the engineering limitations of this approach are becoming increasingly apparent. Taking the state-of-the-art Oriented R-CNN [11] as an example, while its two-stage detection architecture achieves excellent performance on the DOTA remote sensing dataset, it exhibits prohibitively high inference latency when processing aero-engine borescope images. This performance falls far short of meeting the real-time closed-loop requirements of “detection-decision-action” in civil aviation maintenance. Through in-depth analysis, we identify two primary sources of this efficiency bottleneck: First, the representation of rotated bounding boxes leads to a substantial increase in computational complexity. Second, existing feature extraction networks demonstrate inadequate adaptability to the unique characteristics of aviation images—particularly in their failure to effectively model the spatial relationships between ablation regions and surrounding structures.

To address these critical scientific and technical challenges, this study innovatively proposes an improved YOLOv11 rotation detection framework specifically designed for aero-engine ablation detection. The framework’s novelty primarily manifests in two synergistic technical dimensions: (1) At the feature representation level, we developed a multi-scale context feature learning mechanism based on the C3K2CG module. This module overcomes the limited receptive field constraint of traditional convolutional neural networks by incorporating a context-guided mechanism that enables adaptive fusion of local features with surrounding contextual information. Experimental results demonstrate this design’s effectiveness in capturing contextual features across different scales. (2) For computational efficiency optimization, we innovatively implemented a decomposed convolution strategy through the C2PSLA module. By decomposing conventional 2D convolution kernels into sequential horizontal and vertical 1D convolution kernels, this module maintains feature extraction capability while significantly reducing the additional computational overhead induced by rotated bounding boxes, with the added benefit of slightly improved detection accuracy.

Experimental results on our proprietary aero-engine ablation dataset show that the improved model achieves 78.5% mAP@0.5:0.95, representing a 4.2% improvement over the YOLOv11-obb model which model without the specialized data augmentation. This provides an efficient and precise solution for aero-engine ablation detection.

## 2. Related Work

In the domain of aero-engine defect detection, early research primarily relied on image processing techniques. Zheng et al. [12] proposed a morphology-based defect detection method employing erosion and dilation operations to extract defect contours, demonstrating effective performance in magnetic tile surface defect inspection. Similarly, Zou et al. [13] implemented a real-time flaw segmentation approach using optimized Gabor filter banks (retaining only horizontal and vertical orientations), achieving computational efficiency in fabric defect detection. While these methods could identify 0.2 mm-level cracks, their performance heavily depended on controlled imaging conditions, with false detection rates increasing significantly when oil stains or reflections were present. The application of deep learning has substantially improved aircraft engine defect detection capabilities. Yan et al. [14] achieved 95.36% accuracy in aero-engine component defect classification using a modified ResNet-18 network, addressing data scarcity through augmentation (rotation, cropping) and adapting fully connected layers for six-class classification. Wu et al. [15] developed the Rotation Dense Attention Network (RDA-Net), integrating deformable convolution with channel attention mechanisms to enhance rotational robustness by 40% while attaining 85.6% mAP in blade damage detection. Recent advancements have focused on multi-sensor data fusion. Huang et al. [16] proposed a deep Boltzmann machine (DBM)-based multimodal fusion framework that jointly models sensor data with physical simulation information, achieving 98.8% accuracy in aero-engine fault diagnosis. This approach innovatively employed Kullback–Leibler divergence to quantify performance degradation trends, significantly outperforming single-modality models. However, such systems require complex calibration procedures and struggle to meet real-time requirements for onboard equipment.

Currently, there are three main methods for rotated object detection: anchor-based adjustment methods, angle prediction-based methods, and key point detection-based methods.

The anchor-based adjustment approach extends traditional detectors’ anchor mechanisms by adding a rotation angle θ (typically defined as the angle with the positive *x*-axis) to the original horizontal anchor parameters (x, y, w, h), forming a five-parameter representation (x, y, w, h, θ). During feature extraction, deformable convolution learns rotation-sensitive features, while the Region Proposal Network (RPN) presets multi-angle anchor templates and refines parameters through regression branches. Angle prediction commonly employs sliding window classification, discretizing continuous angle space into K intervals (e.g., every 5°). Ma et al. [17] proposed RRPN, which first introduced rotated anchors into the Faster R-CNN framework, achieving 72.5% mAP on the DOTA dataset through rotated IoU calculation for region proposals. Qian et al. [18] further developed the R-DFPN network, using angle-sensitive convolutional kernels to enhance rotated feature representation, improving ship detection accuracy to 78.3%. However, these methods typically have 2–3 times higher computational complexity than horizontal detectors [19] and show limitations when handling extreme aspect ratio aviation targets (e.g., wings with span ratios >10:1).

The angle prediction-based method directly adds an angle prediction branch to single-stage detectors (e.g., the obb module in the YOLO series). Yang et al. [20] proposed CSL-YOLO, innovatively applying circular smooth labels to angle classification, solving the periodic jump problem in traditional regression methods. However, experiments show that when target rotation angles exceed 60°, the localization error of such methods increases by 1.8 times [21].

The key point detection-based method eliminates anchor mechanisms by predicting target center heatmaps and then regressing offsets (Δx, Δy) from the center to the four vertices of the rotated box. This parameterizes the rotated box as a polar coordinate representation (center point, major axis radius, minor axis radius, principal axis angle), with independent feature channels predicting location, scale, and angle information. Law et al. [22] pioneered the key point-based rotated detection paradigm with CornerNet. However, key point methods are sensitive to feature map resolution, with performance significantly degrading when target pixel areas are <32 × 32 [23].

**Figure 2:**
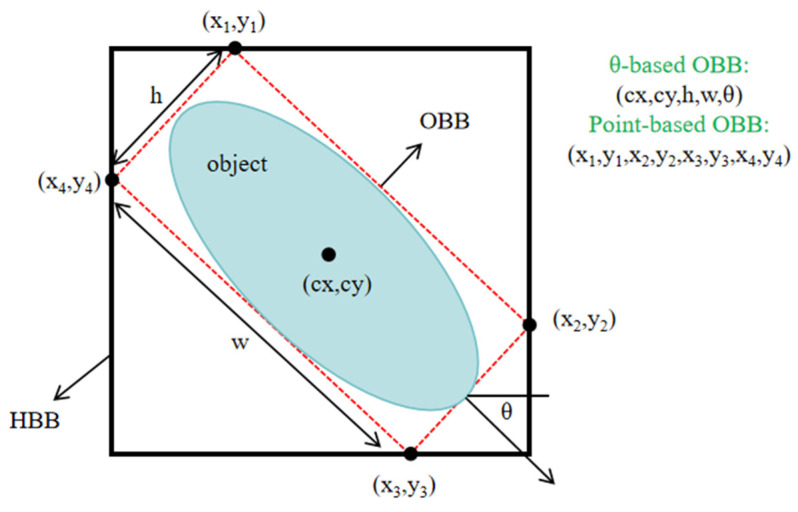
Rotated Bounding Box Annotation Structure, with two representations: (x, y, w, h, θ) and (x1, y1, x2, y2, x3, y3, x4, y4). In the image, the black box represents the horizontal detection target box, the red box represents the rotated detection target box, and the blue area represents the actual target area. w represents the width of the rotated detection box, h represents the height of the rotated detection box, and θ represents the rotation angle of the box. (cx, cy) represents the center point of the target, and (x_i_, y_i_) represents the coordinates of a point.

YOLOv11 represents a recent advancement in the YOLO series architecture for object detection, released in 2024. While maintaining the real-time efficiency characteristic of the YOLO family, it significantly enhances detection performance through deformable convolution, adaptive feature selection mechanisms, and innovative self-attention augmentation modules. Unlike traditional horizontal bounding box detection, rotated object detection provides more precise localization and description for non-horizontally aligned targets. As illustrated in Figure 2, rotated bounding box representations primarily employ two formats: the angle-offset-based representation (x, y, w, h, θ) and the vertex-coordinate-based representation (x1, y1, x2, y2, x3, y3, x4, y4). Compared to the angle-offset approach, the vertex-coordinate method directly describes the geometric shape of rotated boxes without angle boundary issues, making it particularly suitable for irregular or non-rectangular rotated objects. Liu et al. [24] enhanced the YOLOv8 rotated detection framework by introducing an Enhanced Multi-branch Feature Fusion (EMFF) module and adopting GaussianLoss, achieving notable improvements in satellite remote sensing image detection accuracy.

In recent years, with the widespread application of deep learning in computer vision, lightweight network design has gained increasing attention in object detection [25]. CGNet (Context-Guided Network) [26], originally proposed for semantic segmentation tasks, features its core innovation in the Context-Guided Block (CGB). This module combines local feature extraction with multi-scale context information fusion, significantly enhancing feature representation capability while maintaining low computational complexity. In semantic segmentation benchmarks like Cityscapes, CGNet achieved accuracy comparable to more complex models while preserving real-time inference speed [27]. Inspired by CGNet’s excellent performance in segmentation tasks, researchers began exploring its core concepts for object detection. Wang et al. [28] introduced the context-guided mechanism into RetinaNet, significantly improving small object detection performance. These studies demonstrate that appropriate incorporation of contextual information can compensate for traditional CNNs’ limitations in long-range dependency modeling.

Recently, large-kernel convolution has shown growing advantages in computer vision tasks. However, applying standard large-kernel convolution leads to quadratic growth in computational complexity [29]. To address this, Lau et al. [30] improved the LKA module and proposed LSKA (Large Separable Kernel Attention). By decomposing large convolution kernels into spatially separable convolutions, the LSKA module effectively reduces computational complexity while preserving the benefits of large receptive fields. Most importantly, this module innovatively combines large-kernel convolution with attention mechanisms. Chen et al. [31] achieved 1.2% higher accuracy than ConvNeXt with fewer parameters on ImageNet classification by replacing traditional large-kernel convolution with the LSKA module.

## 3. Methodology

### 3.1. Data Processing

In defect detection tasks, random data augmentation techniques—such as random scaling, cropping, and flipping—are commonly employed to expand training datasets. However, relying solely on such random augmentation methods may introduce nearly duplicate samples and result in the loss of critical edge and texture details, ultimately leading to model overfitting and limited generalization capability.

To address these challenges, this study implements a comprehensive preprocessing pipeline. Each original image is individually processed through a suite of data augmentation techniques, including random flipping, random rotation, Mixup, Mosaic, Gaussian filtering, and histogram equalization. Subsequently, both the original image and all its augmented counterparts undergo Z-Score normalization. This process yields seven distinct images from a single original input. First, six data augmentation strategies are employed: random flipping, random rotation, Mixup, Mosaic, Gaussian filtering and histogram equalization. The applied data augmentation strategies target different aspects of model performance. Random flipping and rotation enhance the model’s robustness to changes in object scale, position, and orientation by altering the geometric structure of the input. This capability is particularly crucial in the field of engine inspection, as visible ablation areas dynamically change with the movement of the inspection probe. Learning from such augmented data enables the model to develop the ability to remain unaffected by the rotation angles of objects. Meanwhile, Mixup and Mosaic improve the model’s generalization ability and overall robustness by synthesizing complex training examples from multiple images, thereby encouraging the model to learn broader and more context-rich features. In Mixup, two images are randomly scaled and blended into one composite image, with corresponding adjustments to label coordinates. Mosaic combines four randomly selected images after scaling, arranging them in top-left, bottom-left, top-right, and bottom-right quadrants to form a single image. Gaussian filtering is an image blurring technique that convolves the image with a Gaussian kernel. The degree of blurring is controlled by a tunable parameter, the kernel size/standard deviation (σ); larger values of σ result in a more pronounced blur effect. This technique can simulate real-world blurring caused by factors like defocus or object motion, thereby enhancing the model’s generalization capability. Histogram equalization redistributes pixel intensity values to enhance contrast in darker regions and moderate brightness in overexposed areas, resulting in a more uniform grayscale distribution. For the six augmentations mentioned above, the labels for the bounding boxes with orientations were also correctly transformed for each augmentation. These augmentation methods collectively increase sample diversity, mitigate overfitting, and improve model generalization. Finally, Z-Score normalization is applied to transform the image data distribution to a standard normal distribution with zero mean and unit variance, facilitating faster model convergence and enhanced training stability.

### 3.2. Architecture

**Figure 3:**
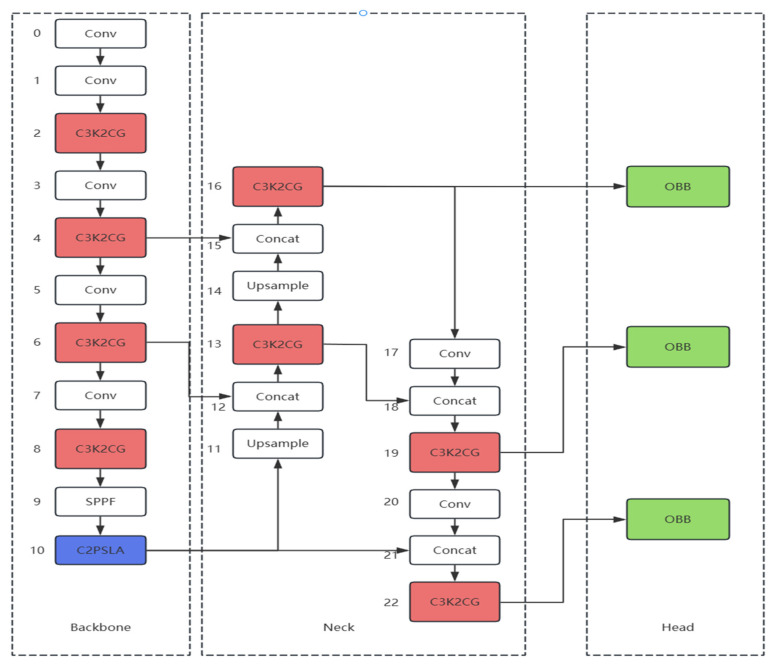
The model architecture of CLO-YOLOv11. The Backbone is used to extract multi-level feature maps from the input image, the Neck efficiently fuses and enhances features from different layers of the backbone network, and the Head uses the optimized feature maps from the neck for dense predictions. The numbers in the figure represent the layer numbers. The layers with white boxes are the original YOLOv11 layers, which have not been modified. The layers with red boxes indicate that the original C3K2 modules have been replaced with C3K2CG modules. The layers with blue boxes indicate that the original C2PSA modules have been replaced with C2PSLA modules. The layers with green boxes indicate that the detect heads for horizontal detection have been replaced with the official OBB detection heads specifically designed for rotation detection.

Compared to the baseline YOLOv11-obb model, this study enhances detection performance through two key structural improvements in the network architecture. As shown in Figure 3, the overall network structure of CLR-YOLOv11 comprises three fundamental components: Backbone, Neck, and Head. In both the Backbone and Neck components, the original convolutional modules have been replaced with context-guided modules (C3K2CG), enabling the model to focus more effectively on critical regions while suppressing irrelevant background interference, which significantly improves the detection capability for small targets. Subsequent experimental validation demonstrates that replacing all C3k2 modules with C3K2CG modules yields the maximum performance improvement. Additionally, the last layer of the Backbone has been replaced with a separable large-kernel attention mechanism module, which effectively expands the module’s receptive field while optimizing computational efficiency, with the Head component utilizing an OBB module. The input RGB images first undergo histogram equalization to enhance brightness and remove noise, followed by Z-Score normalization for standardized processing. The processed images are then fed into the backbone network for contextual feature extraction, where the attention mechanism further enlarges the receptive field and reduces computational complexity. Finally, the feature maps are used by the rotated object detection head for target detection. The official default types for the Intersection over Union (IoU) loss function and the Non-Maximum Suppression (NMS) algorithm were retained as provided, with no deliberate alterations made in this experimental setup.

#### 3.2.1. Context-Guided Module

**Figure 4:**
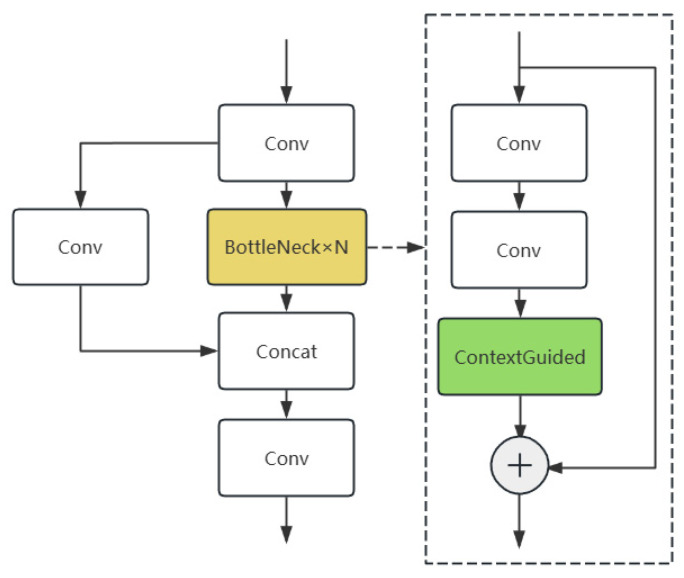
C3K2CG Module Architecture. It achieves contextual feature fusion by introducing the Context-Guided Block in the Bottleneck structure. The yellow boxes indicate modified modules, and the green boxes indicate added modules.

YOLOv11, as a representative single-stage detector, achieves a good balance between real-time performance and detection accuracy. However, its backbone network still primarily relies on local convolutional operations, exhibiting limited capability in modeling global contextual information in complex scenarios. Particularly when processing small targets, the lack of effective contextual information integration leads to degraded detection performance. To address this limitation, this study incorporates a Context-Guided Convolutional Network (C3K2CG) into the feature extraction backbone. Specifically, the original YOLO’s Bottleneck structure is redesigned into a three-stage processing pipeline: first performing channel compression through 1 × 1 convolution, then extracting spatial features using 3 × 3 convolution, and finally introducing the core component of CGNet (the Context-Guided Block) to achieve contextual feature fusion (see Figure 4).

**Figure 5:**
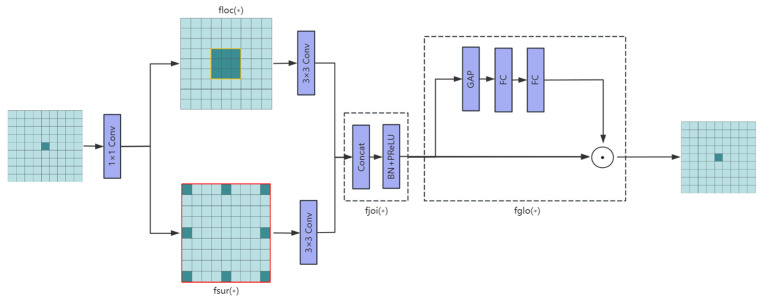
Context-Guided Block Architecture. The ‘∗’ in the diagram represents the data passed from the previous module. In the figure, green represents the incoming image data, dark green represents the module’s processed data. Starting from a small central pixel, floc uses a standard 3 × 3 convolutional layer to precisely capture fine-grained interactions between the central pixel and its eight neighboring pixels, while fsur uses another 3 × 3 convolutional layer to capture interactions among the 9 pixels within a 9 × 9 area around the central pixel. Afterwards, through a series of modules, it is finally transformed back into a single pixel. The blue boxes represent the modules.

Figure 5 illustrates the architecture of the Context-Guided Block module, whose workflow consists of two tightly coupled processing stages. In the first stage, the module employs a dual-path parallel structure to simultaneously extract spatial features at different granularities: the local feature path (floc) utilizes a standard 3 × 3 convolutional layer to precisely capture fine-grained interactions between center pixels and their eight-neighborhood pixels, focusing on detailed characteristics like textures and edges, while the surrounding context path (fsur) employs another 3 × 3 convolutional layer to model broader semantic context within the same receptive field, creating complementary representations. These dual-path features are subsequently integrated through a feature fusion module (fjoi) that first concatenates them along the channel dimension, then employs batch normalization (BN) to stabilize the training process, and finally applies parametric ReLU (PReLU) activation to introduce learnable nonlinearity (Equation (1)), The second stage processes the fused features through a global context modeling module (fglo), typically implemented via global average pooling followed by 1 × 1 convolution to establish long-range channel-wise dependencies, where global statistics serve as attention weights to adaptively enhance local features. The entire processing flow incorporates residual connections that sum the original input with the enhanced features, preserving both low-level details and integrated multi-level context to ultimately output feature maps with rich semantic representation capabilities.

(1) $$PReLU(x)=\left\{\begin{array}{cc} x & if\;x\geq 0\\ a_{i}x & if\;x<0 \end{array}\right.$$

here, x is the input value, and a_i_ is a learnable parameter that controls the slope of the negative value region. When a_i_ = 0, PReLU degenerates to the standard ReLU function; when a_i_ is a fixed small positive number, it is similar to Leaky ReLU.

#### 3.2.2. Separable Large-Kernel Attention Mechanism Module

**Figure 6:**
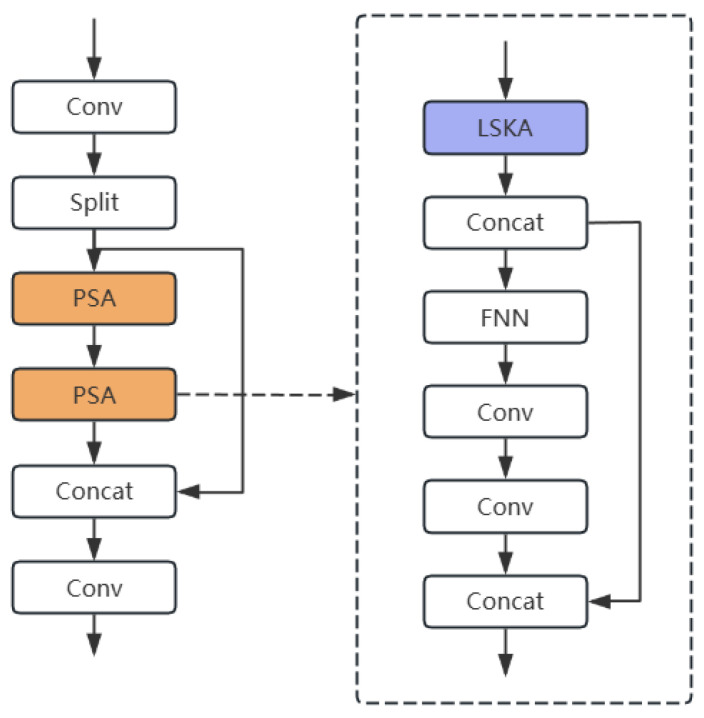
C2PSLA Module Architecture. By adding the LSKA module before the PSA module, the effective receptive field is expanded, and computational efficiency is optimized. The yellow boxes indicate modified modules, and the blue boxes indicate added modules.

Although the C2PSA module in YOLOv11 enhances positional awareness through coordinate information, its reliance on 3 × 3 convolution kernels results in a limited receptive field, making it difficult to effectively capture global contextual information from large-scale targets. By integrating the LSKA module with the C2PSA module, we not only expand the effective receptive field but also optimize computational efficiency (see Figure 6). Specifically, this integration replaces the attention mechanism in the original PSA component of the C2PSA module with the LSKA mechanism.

**Figure 7:**
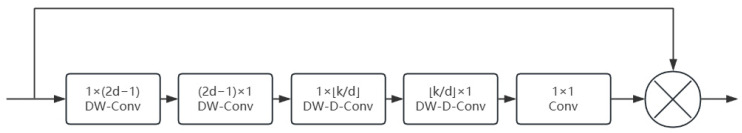
LSKA Module Architecture. First, lightweight feature extraction is performed through small kernel convolution, and then the receptive field is expanded via dilated convolution. Both processes involve progressive feature processing using separable convolutions in the horizontal and vertical directions.

The proposed LSKA module processes input feature maps through a carefully designed sequence of operations that balance receptive field size and computational efficiency. As shown in Figure 7, the input feature map x is first duplicated as the original signal u to preserve untreated information for subsequent residual enhancement. The LSKA module then performs progressive feature processing using separable convolutions in horizontal and vertical orientations. The initial stage employs small-kernel convolutions (e.g., 1 × 3 and 3 × 1) for lightweight feature extraction, where these directional convolutions operate independently along horizontal and vertical axes through group convolution (groups = dim) to maintain channel-wise processing efficiency. The module then progresses to the large-kernel attention computation phase, which utilizes dilated convolutions with larger kernels (e.g., 1 × 11 or 1 × 17) to expand the receptive field. These horizontal and vertical convolutions execute sequentially but employ larger dilation rates (e.g., dilation = 2 or 3) to achieve global attention-like coverage. Notably, all large-kernel convolutions retain channel grouping properties to ensure manageable parameter counts. Every convolutional layer implements zero padding to preserve spatial resolution throughout the processing pipeline.

Following the aforementioned convolutional sequence processing, the feature maps undergo channel mixing and weight calibration through a 1 × 1 convolution, which functions similarly to the projection layer in attention mechanisms by fusing multi-scale features into a unified attention map. Ultimately, the LSKA module employs multiplicative interaction to perform element-wise multiplication between the generated attention map and the original feature map u, achieving feature re-weighting. This design preserves the dynamic adjustment capability of standard attention mechanisms while enhancing stability through the inductive bias of convolutions. Throughout the entire process, all convolutional operations maintain consistent input and output spatial dimensions, facilitating seamless integration into various network architectures. By adopting this decomposed large-kernel convolution strategy, LSKA achieves long-range dependency modeling comparable to global attention mechanisms while avoiding computational explosion, making it particularly well-suited for high-resolution vision tasks. The output of LSKA can be derived using the following formula:

(2) $${\bar{z}}^{C}=\sum_{H,W}W_{\left(2d-1\right)\times 1}^{C}\times (\sum_{H,W}W_{1\times \left(2d-1\right)}^{C}*F^{C})$$

(3) $$Z^{C}=\sum_{H,W}W_{\lfloor \frac{k}{d}\rfloor \times 1}^{C}\times (\sum_{H,W}W_{1\times \lfloor \frac{k}{d}\rfloor }^{C}*{\bar{z}}^{C})$$

(4) $$A^{c}=W_{1\times 1}\times Z^{C}$$

## 4. Experiments and Results

### 4.1. Data Collection

**Figure 8:**
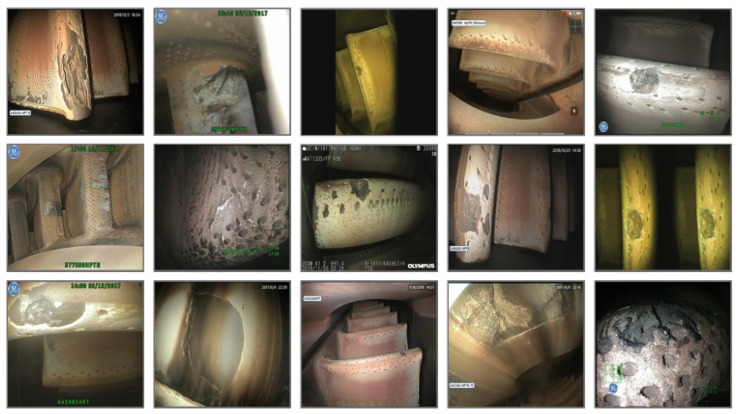
Representative Samples from the Original Dataset. This not only includes turbine blade erosion, but also combustion chamber erosion and nozzle/tailpipe erosion. The boxes, text, icons, etc. in the figure are displays of the instrument during testing; they have no effect on the experiment and do not affect the experiment.

The aero-engine ablation dataset utilized in this experiment consists of 805 original images with a resolution of 480 × 640 pixels, as partially illustrated in Figure 8. Given the limited scale of the original dataset, six data augmentation strategies—random flipping, random rotation, Mixup, Mosaic, Gaussian filtering and histogram equalization—were employed to expand the sample size, enhance diversity, and mitigate model overfitting. Due to the overall low brightness of the captured images, all augmented data underwent Z-Score normalization to improve image luminance and reduce noise, thereby facilitating more effective feature learning by the model while simultaneously enhancing image quality to boost both training performance and generalization capability. After processing, the final dataset comprised 5614 images. Following standard practice, the dataset was partitioned into training, validation sets at an 8:2 ratio during the training process. For the comparative models in Section 4.4, the data augmentation was limited to six methods—random rotation, horizontal flip, vertical flip, random translation, random crop, and random scaling—to ensure that the training performance was not influenced by the size of the training set. This approach maintained the total number of input images at 5614 for all models. For rotated object detection, this experiment employs a four-point annotation format, denoted as (x1, y1, x2, y2, x3, y3, x4, y4). This same format is used for the comparative models, including R-Faster R-CNN, R3Det, and S2ANet.

### 4.2. Experimental Environment

The experiments were conducted in a virtual environment configured with PyTorch 2.0.0 + cu118 and Python 3.10, running on a hardware system consisting of an Intel(R) Core(TM) i7-14700K CPU, 64 GB RAM, and an NVIDIA GeForce RTX 4090 GPU with 48 GB memory, using the Windows 11 Professional operating system. All models training employed the following parameter configuration: 200 epochs with a batch size of 64, YOLO series’ input images uniformly resized to 640 × 640 pixels, other models’ input images uniformly resized to 1024 × 1024 pixels, 8 worker threads for data loading, and no pretrained weights utilized, network optimization was performed using the SGD optimizer with a learning rate of 0.01 for weight updates, using L2 regularization with a value set to 0.001. All models also employed early stopping, stopping training if the validation loss did not improve at all over 10 consecutive epochs (with a threshold of 0.001). All models were able to stop during the 200 epochs of training. The other parameters are set to the model’s default values. All baseline models are trained in the same way and do not use pre-trained weights.

### 4.3. Evaluation Metrics

The evaluation metrics for rotation detection and horizontal detection are calculated in the same way, but the calculation method for IoU is different. The primary evaluation metrics adopted in this study include precision (P), recall (R), F1-score and mean average precision (encompassing both mAP@0.5 and mAP@0.5:0.95), all derived from the confusion matrix with the following mathematical formulations:

(5) $$P=\frac{TP}{TP+FP}$$

(6) $$R=\frac{TP}{TP+FN}$$

(7) $$AP=\int_{0}^{1}P\left(R\right)dR$$

(8) $$mAP=\frac{1}{k}\sum_{i=1}^{k}AP$$

(9) $$F1-score=\frac{2PR}{\left(P+R\right)}$$

TP indicates predicted boxes with IoU above threshold versus ground truth, FP denotes predicted boxes with IoU below threshold, and FN represents missed ground truth objects. mAP@0.5 is the mean AP across all classes at IoU = 0.5. mAP@0.5:0.95 calculates each class’s average AP over IoU thresholds from 0.5 to 0.95 (step 0.05), and then averages across classes.

### 4.4. Comparative Experiments

To validate the superiority of the proposed CLO-YOLOv11 model, this experiment incorporates comparisons not only against other models from the YOLO series but also against three contemporary models specifically designed for rotated object detection: Rotated Faster R-CNN, R^3^Det, and S2ANet. Faster R-CNN is a foundational work for two-stage object detectors, whose core innovation is the Region Proposal Network (RPN). The RPN integrates the candidate region generation step into an end-to-end neural network, significantly improving both speed and accuracy. R^3^Det achieves more precise localization in dense object scenarios through cascaded refinement. It introduces a Feature Refinement Module (FRM) that progressively adjusts the feature maps to achieve better alignment with the refined rotated bounding boxes. S2ANet focuses on achieving efficient and accurate feature alignment within a single-stage framework. Its core design is the Align Convolution Module, which adaptively convolves features based on the angle and size of the rotated bounding boxes, ensuring that the extracted features precisely match the orientation of the targets. All experimental results presented in Table 1.

Empirical data from our evaluation of the YOLO series (v5, v8, v11, v12) for horizontal detection validates the overall superiority of YOLOv11, with it attaining an accuracy of 88.4% and an mAP@0.5:0.95 of 68.8%. An additional critical finding pertains to training efficiency: YOLOv11 converges fastest, while YOLOv12 demands a computational cost more than double that of its counterparts. Therefore, we conclude that implementing rotated detection on the YOLOv11 architecture is justified to achieve optimal performance, balancing both high accuracy and lower resource consumption.

The comparison between YOLOv11-obb and YOLOv11 demonstrates that simple architectural modifications in YOLOv11-obb yield significant performance improvements. Compared to the baseline YOLOv11, this model only replaces the conventional detect module in the final detection head with a specially designed OBB module, yet it achieves comprehensive performance breakthroughs. Specifically, precision shows a substantial 2.7% increase from 88.4% to91.1%, indicating effective control of false positives while maintaining high recall. Recall rate also improves by 5.4 percentage points to 90.4%, enhancing the model’s ability to capture genuine defects. Regarding comprehensive performance metrics, mAP@0.5 increases by 4.1% to reach 91.8%, while the more challenging mAP@0.5:0.95 metric also improves by 5.5%, demonstrating more stable performance across different IoU thresholds.

The performance enhancement primarily stems from the precise adaptation of rotated bounding boxes to the unique morphology of aircraft engine blades. Conventional horizontal rectangular boxes exhibit inherent limitations when detecting defects in slender, obliquely arranged blades, often introducing significant background noise or causing localization inaccuracies. The obb module employs a four-point representation that enables tight alignment with the actual contours of blades and defect areas. This improvement proves particularly crucial for ablation detection tasks: first, the precise point positioning allows better differentiation between adjacent blade edge regions, reducing false detections; second, the rotated boxes provide superior encapsulation of directional features like oblique cracks, thereby improving the complete detection rate of defects.

A comparison between YOLOv11-obb and three classical rotated object detection models reveals that Rotated Faster R-CNN, R3Det, and S2ANet perform poorly on our dataset. The primary reason may lie in the fact that YOLOv11-obb, as a single-stage detector, possesses a relatively simple architecture and demonstrates excellent data utilization efficiency. This enables it to achieve better generalization on limited data while avoiding overfitting. In contrast, the two-stage mechanism of Faster R-CNN and the cascaded refinement structure of R3Det involve more parameters and a more complex pipeline. When the dataset is insufficient, these models are more prone to overfitting, making it difficult to realize their theoretical accuracy advantages. From the perspective of target characteristics, although ablation regions exhibit irregular shapes that require rotated bounding boxes for compact annotation, their appearance variation and density are generally not as extreme as those of targets like aircraft or ships in remote sensing images. The core innovations of R3Det and S2ANet—such as the Feature Refinement Module and Aligned Convolution—are primarily designed to address strict feature alignment for highly dense, arbitrarily oriented objects. For ablation region detection, the marginal accuracy gain brought by these complex mechanisms may be insignificant. Instead, their complexity increases the risk of training failure on insufficient data. The single-stage, straightforward design of YOLOv11-obb can effectively learn the overall features of ablation regions and is already sufficient to meet the localization accuracy requirements in most cases.

### 4.5. Ablation Study

To systematically evaluate the effectiveness of each newly introduced module and the specialized data augmentation strategy—which involves applying six augmentations (random flipping, random rotation, Mixup, Mosaic, histogram equalization, and Gaussian filtering) to the original dataset followed by Z-score normalization—we conducted a comprehensive ablation study. The experimental results are presented in Table 2.

- Model 1 is the baseline YOLOv11-obb model without the specialized data augmentation. To maintain the same dataset size, it employs six augmentation methods: random flipping, vertical flipping, horizontal flipping, random scaling, random cropping, and random translation, ensuring the input dataset contains 5614 images—the same as that of CLO-YOLOv11.
- Model 2 is a CLO-YOLOv11 model using an alternative data augmentation approach: first applying CLAHE to the original dataset, followed by six augmentations—random rotation, random flipping, random translation, Mixup, Mosaic, and Gaussian filtering—and concluding with Z-score normalization.
- Model 3 is another CLO-YOLOv11 variant with an alternative augmentation strategy, differing from Model 3 by replacing Gaussian filtering with random cropping.
- Model 4 is the YOLOv11-OBB model enhanced with the specialized data augmentation strategy.
- Model 5 builds upon YOLOv11-OBB by incorporating both the specialized data augmentation and the C3K2CG module.
- Model 6 enhances YOLOv11-OBB with the specialized data augmentation and the C2PSLA module.
- Model 7 is the proposed CLO-YOLOv11 model in this study, which integrates the specialized data augmentation, C3K2CG module, and C2PSLA module into the YOLOv11-OBB architecture.

Comparative analysis between Model 1 and Model 4, as well as among Models 2, 3, and 7, clearly demonstrates the effectiveness of the specialized data augmentation pipeline proposed in this study. Model 4 achieves a 1.4% improvement in precision and a 3.2% increase in mAP@0.5:0.95 compared to Model 1. Although a slight decrease in recall is observed, this may be attributed to the more complex and challenging training samples created by our augmentation strategy, which leads to more reliable yet conservative predictions. Furthermore, the comparison among Models 2, 3, and 7 indicates that incorporating CLAHE does not enhance model performance but rather results in marginally inferior results.

Through a comparative analysis between Models 4, 5, 6, and 7, the experimental results demonstrate that each added module contributes to performance improvement. The incorporation of the C3K2CG module yields a 0.9% increase in precision, 0.2% increase in F1-score and a 0.9% improvement in mAP@0.5:0.95, while the C2PSLA module enhances precision by 0.9%, recall by 0.3%, and mAP@0.5:0.95 by 0.9%. The complete Model 7, integrating both modules, achieves further gains over Models 5 and 6, ultimately delivering a 1.4% precision improvement, 0.3% recall increase, 0.8% mAP@0.5 boost, 1.0% mAP@0.5:0.95 enhancement and 0.9% F1-score improvement compared to Model 4. Detailed analysis reveals complementary functionality between these modules: the C3K2CG module strengthens spatial context awareness through its gating mechanism, while the C2PSLA module optimizes information flow paths in the feature pyramid. This synergistic combination explains the progressive performance improvement observed across the ablation study variants.

**Figure 9:**
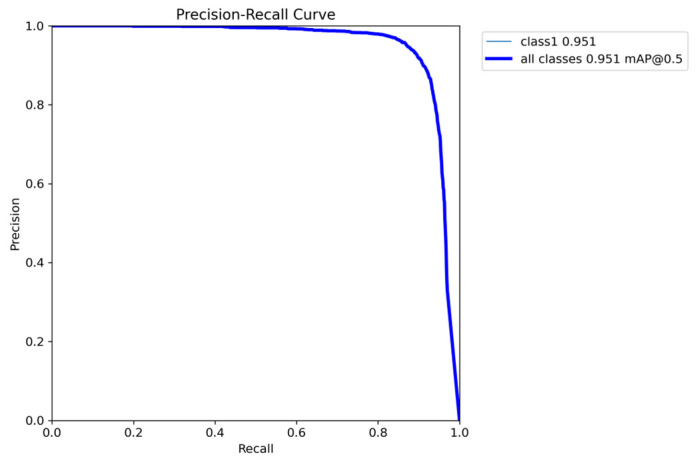
PR curve of CLO-YOLOv11. The purpose of the experiment is to detect ablation areas, with all ablation types set as class 1, therefore, the light blue and dark blue lines overlap. The mean average precision (mAP) at an IoU threshold of 0.5 reaches 0.951.

Figure 9 presents the Precision–Recall curve of CLO-YOLOv11, which is used to evaluate the performance of the object detection model. The curve shows that for class1 (detecting only the ablation area without distinguishing ablation categories), the mean Average Precision (mAP) at an IoU threshold of 0.5 reaches 0.951, indicating exceptionally high detection accuracy. The curve commences from a point of 1.0 precision and 0.0 recall, and as recall increases, precision remains at a high level. The overall curve is smooth and remains close to the upper-right corner, reflecting the model’s ability to maintain high precision across varying recall rates and demonstrating outstanding robustness.

Table 3 presents the parameter counts for Models 4, 5, 6, and 7. The average detection times presented in the table were obtained under the following rigorous testing conditions: all timings were performed on an RTX 4090 GPU and represent the total end-to-end latency. This includes data preprocessing, the model’s forward pass, and the complete post-processing pipeline, which comprises decoding, confidence score filtering, and the essential rotated Non-Maximum Suppression (NMS) operation. The evaluation was carried out with a fixed batch size of 32 and in FP32 precision. Comparative analysis between C3K2CG-YOLOv11-obb and YOLOv11-obb reveals that the former not only reduces model parameters but also improves detection speed. The complete CLR-YOLOv11 achieves enhanced performance with only an 11% parameter increase while maintaining comparable detection speed.

Based on the computational complexity analysis of the CLO-YOLOv11 model under a 640 × 640 input resolution, the distribution characteristics of computational load across different stages can be clearly observed. The Backbone feature extraction stage accounts for over half of the total computations, primarily due to the dense convolutional layers and residual connections within its architecture, which constitute the main computational burden of the model. The Neck feature fusion stage contributes approximately 20% of the computations, with its high-channel-count feature maps leading to a significant computational load. The OBB detection head stage occupies only 10% of the total FLOPs; it is mainly responsible for generating rotated bounding box parameters and classification confidence scores, and although it represents the smallest proportion of computations, it directly determines the specialized output format required for OBB tasks.

**Figure 10:**
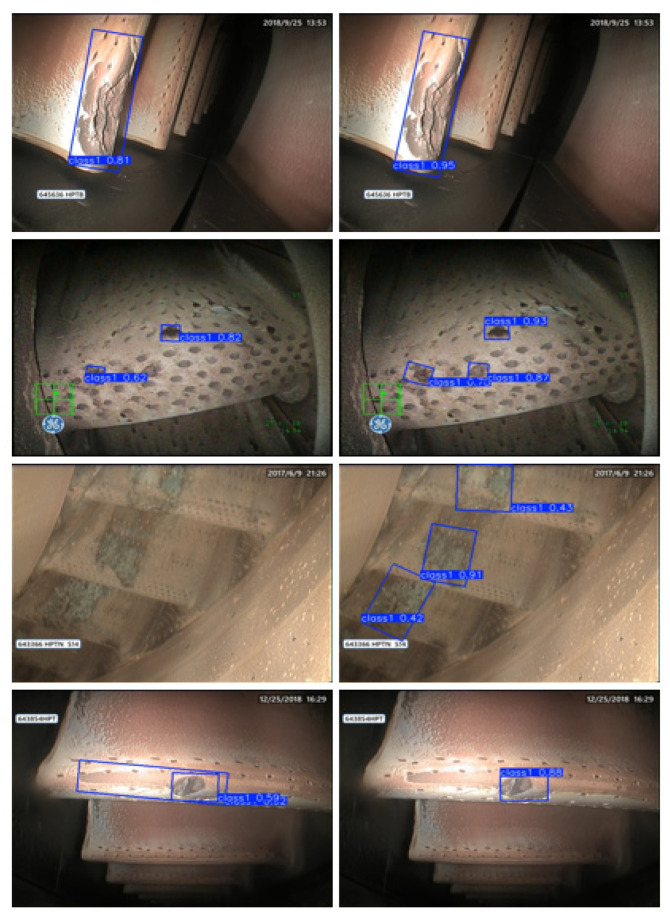
Comparative Visualization of Detection Results between YOLOv11-obb and CLR-YOLOv11. The first column shows the detection results of the YOLOv11-obb model without using special data augmentation on some original images, while the second column shows the detection results of the complete CLO-YOLOv11 model on the same images. The boxes, text, icons, etc. in the figure are displays of the instrument during testing; they have no effect on the experiment and do not affect the experiment.

Figure 10 visually demonstrates the detection results comparison between YOLOv11-obb and the improved CLR-YOLOv11 on aero-engine ablation cases. In the first image, CLO-YOLOv11 demonstrates superior detection confidence compared to YOLOv11-OBB. In the second and third images, CLO-YOLOv11 identifies more ablation areas with higher accuracy than YOLOv11-OBB. In the fourth image, YOLOv11-OBB produces a false positive detection by marking a non-existent area. The enhanced model exhibits more precise bounding box localization, significantly reduced missed detections, and improved small target detection capability.

## 5. Discussion

### 5.1. Discussion on Model Parameter Selection and Data Augmentation Techniques

**Figure 11:**
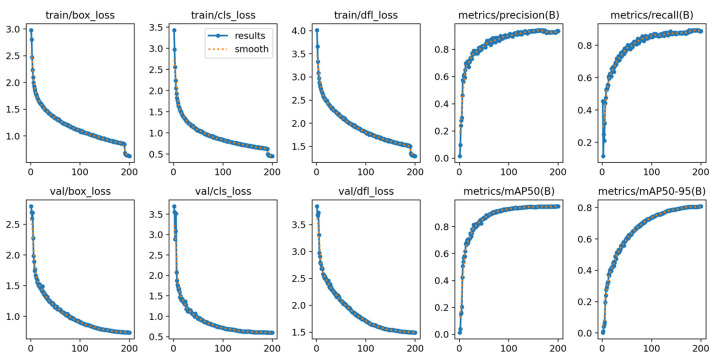
Performance result charts of the CLO-YOLOv11 model without using early stopping. The three charts at the top left are the box_loss, cls_loss, and dfl_loss of the training set, respectively; the three charts at the bottom left are the box_loss, cls_loss, and dfl_loss of the validation set, respectively; the four charts on the right, from top to bottom and left to right, are the accuracy, recall, mAP@0.5, and mAP@0.5:0.95 of the validation set, respectively.

The training epoch number was set to 200 based on repeated experimental validation. Figure 11 presents the training results of the CLO-YOLOv11 model. Both the figure and its corresponding training log files indicate that the model’s loss and performance metrics stabilized after approximately 170 epochs. However, beyond epoch 190, the training loss decreased sharply while the validation loss showed no corresponding improvement, indicating the onset of overfitting. This phenomenon may be attributed to the excessively low learning rate after epoch 190, which likely caused the model to “memorize” the training data rather than continue learning meaningful features. To mitigate this issue, an early stopping strategy was implemented: training was halted if the improvement in validation loss fell below a threshold of 0.001 for 10 consecutive epochs. Experimental results demonstrate that CLO-YOLOv11 stopped training early at around epoch 180, while all other models also completed training within the 200-epoch limit without exhibiting significant overfitting.

Upon conducting multiple rounds of comparative experiments on optimizer selection, we ultimately found that SGD demonstrated superior performance over other optimizers, such as AdamW and Ranger, for the task presented in this paper. The advantage of SGD primarily stems from its better generalization capability. Owing to its inherent stochasticity, SGD tends to converge to flatter minima in the loss landscape—regions that are generally less sensitive to model perturbations, thereby enhancing model robustness on unseen data. In contrast, adaptive optimizers like AdamW and Ranger, despite their faster initial convergence, are prone to converge to sharp minima. While these may yield very low training loss, they often lead to overfitting to noise in the training data, resulting in less stable performance in critical real-world scenarios.

In terms of data augmentation, we initially experimented with basic geometric transformations combined with Mixup and Mosaic. However, experimental results showed limited performance improvement and signs of overfitting. Subsequently, we introduced histogram equalization and Gaussian filtering to enhance dataset complexity and better align with real-world scenarios. While Gaussian filtering was initially introduced for image denoising, experiments revealed that excessive denoising led to image blurring, which instead degraded model performance. Therefore, we repurposed it as a data augmentation technique to simulate image blur in real-world environments, effectively increasing the diversity of data distribution. All augmented images were processed using Z-score normalization. This operation not only standardized the data distribution and improved training stability but also accelerated model convergence. We also systematically compared different preprocessing pipeline sequences (e.g., performing Gaussian filtering and histogram equalization prior to data augmentation and Z-score normalization, or altering the order of these steps). Experimental results demonstrated that the pipeline adopted in this study yielded the best model performance.

### 5.2. Model Interpretability Analysis

**Figure 12:**
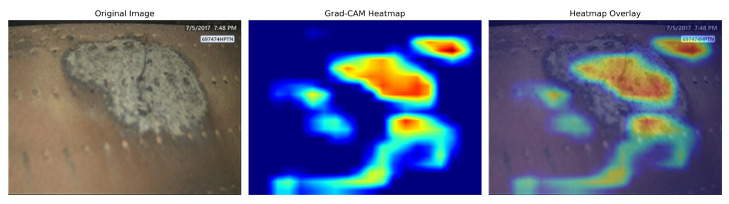
Grad-CAM heatmap of CLO-YOLOv11. The first image is the original image, the second image is the heatmap obtained after Grad-CAM, and the third image is the overlay of the first two images.

To validate the decision logic and reliability of the CLO-YOLOv11 model, we employed Grad-CAM technology to visualize the model’s regions of interest, with the results shown in Figure 12. The visualization clearly demonstrates that the model’s high-activation regions (represented by the red areas in the heatmap) accurately concentrate on the ablation areas of the engine surface in the original image, rather than being uniformly distributed across the entire image or focusing on irrelevant structural backgrounds. This correspondence becomes more intuitive in the heatmap overlay: the model’s attention center shows strong spatial alignment with visually identifiable ablation marks. This indicates that during the decision-making process, the model does not rely on irrelevant noise features but has successfully learned key visual patterns directly associated with “ablation” defects. From an interpretability perspective, this confirms that the detection model proposed in this study possesses good decision transparency and reliability.

### 5.3. Limitations and Future Work

While this study has achieved the expected outcomes, it still exhibits certain limitations in the following aspects.

First, regarding the dataset, although it includes ablation images from various components such as turbine blades, combustion chambers, and nozzles/exhaust nozzles, the diversity in actual engine types and defect morphologies remains insufficient. This may lead to incomplete coverage of certain rare defects or introduce potential data biases. To address this, future research will focus on expanding the dataset scale, incorporating more engine types, and collecting real ablation images under different environmental and lighting conditions, including rare defects, to enhance the diversity and representativeness of the data distribution. Furthermore, the current dataset contains limited common image interference (e.g., smudges, reflections) typically found in real industrial settings. Therefore, the model’s robustness in detecting subtle defects under such complex conditions requires further validation. In the future, we plan to construct more challenging datasets incorporating various interference factors and explore methods such as anti-interference training or domain adaptation to improve the model’s ability to distinguish genuine defects from interference artifacts in complex environments.

Second, in terms of experimental design, this study evaluated the model’s generalization capability and performance only through cross-validation on the RTX 4090 platform. Moving forward, we will validate the model’s generalizability on more diverse datasets involving different materials and mechanical faults. Additionally, we will deploy the model on low-latency embedded systems commonly used in the aviation field (e.g., Jetson, FPGA) to test its inference performance and practical utility in real industrial environments.

## 6. Conclusions

To address the characteristic features of aero-engine ablation images and the precision-efficiency trade-off, this paper proposes an improved YOLOv11 algorithm incorporating C3K2CG and C2PSLA modules. The algorithm achieves 93.9% precision on our proprietary dataset, representing a 2.8% improvement over the YOLOv11-obb model, which is the model without specialized data augmentation, along with a 4.2% increase in mAP@0.5:0.95, meeting the requirements for engineering applications. Although computational load increases slightly, the combined C3K2CG and C2PSLA modules enhance model performance without compromising detection speed.

We acknowledge that the core components of our framework are built upon existing technologies. However, the novelty of this work lies in the problem-oriented redesign and seamless integration of these components into a cohesive system that effectively addresses the unique challenges of aero-engine ablation inspection. The orchestrated data pipeline, the replacement strategy for specific modules, and the achieved balance between accuracy and efficiency are all tailored contributions to this field.

## Figures and Tables

**Table 1:** Comparative Experimental Results. The table includes input image size, accuracy, recall, mAP@0.5, mAP@0.5:0.95, and F1-score.

Model	Input Size	P (%)	R (%)	mAP@0.5 (%)	mAP@0.5:0.95 (%)	F1-Score (%)
YOLOv5	640 × 640	87.1 ± 0.3	82.5 ± 0.4	85.7 ± 0.7	65.5 ± 0.5	84.7 ± 0.2
YOLOv8	640 × 640	88.4 ± 0.4	83.5 ± 1.2	86.4 ± 0.3	67.6 ± 0.5	85.9 ± 0.5
YOLOv11	640 × 640	88.4 ± 0.1	85.0 ± 0.8	87.7 ± 0.4	68.8 ± 1.0	86.7 ± 0.2
YOLOv12	640 × 640	84.5 ± 0.4	81.2 ± 0.5	84.7 ± 0.6	64.9 ± 0.7	82.8 ± 0.4
YOLOv11-obb	640 × 640	91.1 ± 0.2	**90.4** ± 0.4	91.8 ± 0.6	74.3 ± 0.6	90.7 ± 0.2
Rotated-Faster R-CNN	1024 × 1024	-	84.5 ± 0.3	75.9 ± 0.2	-	-
R3Det	1024 × 1024	-	84.6 ± 0.2	78.1 ± 0.2	-	-
S2ANet	1024 × 1024	-	86.4 ± 0.2	79.7 ± 0.3	-	-
ours	640 × 640	**93.9** ± 0.6	88.4 ± 0.6	**94.6** ± 0.4	**78.5** ± 0.5	**91.1** ± 0.5

**Table 2:** Ablation Study Results. The table includes accuracy, recall, mAP@0.5, mAP@0.5:0.95, and F1-score.

Model	P (%)	R (%)	mAP@0.5 (%)	mAP@0.5:0.95 (%)	F1-Score (%)
1	91.1 ± 0.2	90.4 ± 0.4	91.8 ± 0.6	74.3 ± 0.6	90.7 ± 0.2
2	92.4 ± 0.2	85.6 ± 0.8	92.0 ± 0.4	75.5 ± 1.0	88.9 ± 0.2
3	92.8 ± 0.2	86.7 ± 0.6	92.9 ± 0.2	77.6 ± 0.6	89.7 ± 0.3
4	92.5 ± 0.3	88.1 ± 0.8	93.8 ± 0.4	77.5 ± 0.6	90.2 ± 0.5
5	93.4 ± 0.4	87.7 ± 0.3	93.9 ± 0.2	78.4 ± 0.5	90.4 ± 0.3
6	93.4 ± 0.4	88.4 ± 0.6	93.9 ± 0.3	78.4 ± 0.6	90.8 ± 0.4
7	93.9 ± 0.6	88.4 ± 0.6	94.6 ± 0.4	78.5 ± 0.5	91.1 ± 0.5

**Table 3:** Model Performance Comparison. The table includes models’ parameter, computation volume, weights, and average detection time.

Model	Parameter	Computation Volume (GFLOPS)	Weights (MB)	Average Detection Time (ms)
YOLOv11-obb	2,653,918	6.6	7.5	1.3
C3K2CG-YOLOv11-obb	2,995,018	7.9	8.2	1.4
C2PSLA-YOLOv11-obb	2,607,070	6.5	7.4	1.5
CLR-YOLOv11	2,948,170	7.9	8.1	1.4

## Data Availability

The data are not publicly available due to commercial sensitivity and data privacy.
